# Comparisons between Membrane, Bridge and Cantilever Miniaturized Resistive Vacuum Gauges

**DOI:** 10.3390/s120708770

**Published:** 2012-06-27

**Authors:** Kasun Gardiye Punchihewa, Evan Zaker, Rade Kuljic, Koushik Banerjee, Tatjana Dankovic, Alan Feinerman, Heinz Busta

**Affiliations:** Department of Electrical and Computer Engineering, University of Illinois at Chicago, Chicago, IL 60607, USA; E-Mails: kpunch2@uic.edu (K.G.P.); ezaker2@uic.edu (E.Z.); rkulji2@uic.edu (R.K.); kbaner2@uic.edu (K.B.); tdanko2@uic.edu (T.D.); feinerman@uic.edu (A.F.)

**Keywords:** miniaturized vacuum and pressure gauges, MEMS-based Pirani gauges, silicon bulk micromachining, thermal conductivity of metals, insulators, air, low power sensing

## Abstract

Using bulk micromachining, meander-shaped resistor elements consisting of 20 nm Cr and 200 nm Au were fabricated on 1 μm thick silicon nitride membranes, bridges, and cantilevers. The resistance change as a function of pressure depends strongly on the thermal resistance of the two metal lines connecting the heated resistor to the silicon bulk (cold junction) and on the thermal resistance of the silicon nitride. Relative resistance changes ranging from about 3% (small membrane) to 20% (bridge) per mW of input power were obtained when operating the devices in constant voltage mode. The pressure where maximum sensitivity of these gauges occurs depends on the distance ‘d’ between the periphery of the heated resistor element and the silicon cold junction. Devices with ‘d’ ranging from 50 μm to 1,200 μm were fabricated. Assuming that pressures can be reliably measured above the 10% and below the 90% points of the resistance *versus* pressure curve, the range of these devices is about two orders of magnitude. By integrating two devices, one with d = 65 μm and one with d = 1,200 μm on the same chip and connecting them in series, the range can be increased by about a factor of three. By fabricating the cantilever devices so that they curl upon release, it will be shown that these devices also exhibit larger range due to varying ‘d’.

## 1. Introduction

Small vacuum systems are gaining importance for several applications such as micro electromechanical systems-based (MEMS) resonators, RF switches and energy harvesters. These systems have to be able to maintain a given vacuum pressure for extended periods of time. Thus, it is important to be able to monitor pressures inside the vacuum enclosures, preferably at low power. For MEMS resonators and RF switches, the vacuum packages including the active devices and pressure sensors can be fabricated as part of the overall MEMS process [1,2].

Miniaturized pressure sensors include resonating cantilevers, capacitive sensors, microspinning rotor gauges, thermocouple sensors and thermal-based sensors [3–5]. The most popular miniaturized vacuum sensor is the Pirani sensor due to its relative ease of manufacturing, its wide pressure range and low power consumption. It consists of micromachined resistors in single element form (micro wires) or meander-shape. These resistors are free standing or fabricated on thermally insulating supports, such as membranes, bridges or cantilevers. The materials chosen for the resistors elements range from metals such as Al, Au and Ni, to doped polycrystalline silicon. The materials chosen for the thermally insulating platforms are usually silicon dioxide, silicon nitride or a combination of the two. Innovative MEMS surface and bulk micromachining are employed in the fabrication process. As the resistor elements are powered, ranging from several microwatts to milliwatts, they heat up by several degrees near atmospheric pressures, thus increasing their resistance. As the pressure decreases, less gas molecules become available to carry heat away from the resistor elements, thus causing an additional rise in temperature, resulting in increasing resistance. The shape of the resistance-pressure curve resembles an inverted S and is governed by the kinetic gas theory. The position of this inverted S curve along the pressure scale depends strongly on the distance ‘d’ between the heated resistor element and the cold junction, which is usually the substrate. To obtain maximal sensitivity near atmospheric pressures, ‘d’ is about 1 μm. For lower pressure measurements, devices with d's up to several hundred micrometers have been reported. Representative examples of such devices are presented in [6–13].

In this paper, results of three membrane-based Pirani gauges with square-shaped silicon nitride membranes of 470 μm, 1,040 μm and 2,740 μm will be compared with a 420 μm × 1,250 μm silicon nitride bridge device and a 420 μm × 800 μm silicon nitride cantilever device. The resistor elements fabricated on these 1 μm thick silicon nitride support structures are all of the same size with a surface area of 6.3 × 10^−4^ cm^2^ and a line width of 20 μm. It will be shown that by combining two membrane devices with different d's, the pressure range can be increased. A similar result is obtained with the cantilever device which, due to built-in stress gradients in the nitride film, forms an out-of-plane cylinder resulting in a distributed ‘d’ device.

## 2. Device Fabrication

Devices were fabricated on 315 μm thick double side polished, n-type, 3″ diameter (100) silicon wafers with resistivity between 1–10 Ωcm. After growing a 1 μm thick SiO_2_ layer by steam oxidation, a 1.0 μm thick silicon nitride layer was deposited by LPCVD on both sides of the wafer. One side of the wafer was then coated with a 1.5 μm thick layer of Shipley S1818 positive photoresist. This was followed by photolithography to define the meander-shaped resistors. After pattern definition, a 20 nm thick Cr layer was deposited followed by a 200 nm thick gold layer by e-beam evaporation. Lift-off in acetone completed the resistor definition. Next, backside lithography was performed to define square openings in the silicon nitride layer for KOH etching of bulk silicon. A third photolithographic step was performed to define the outlines of the bridges and cantilevers on the top surface. A plasma etching step in a mixture of CF_4_ and 4% O_2_ was performed next to etch the unwanted silicon nitride layers on the front and back of the wafer. A protective layer (Brewer Science's ProTEK B3) was then applied to protect the top surface while the backside of the substrate was etched in buffered HF to remove the remaining silicon dioxide that was not etched in the plasma etching step followed by KOH etching until the silicon dioxide/silicon nitride membranes, bridges and cantilevers were exposed. The silicon dioxide at the front of the wafer was then removed in buffered HF followed by the removal of the protective layer in acetone.

**Figure 1.**
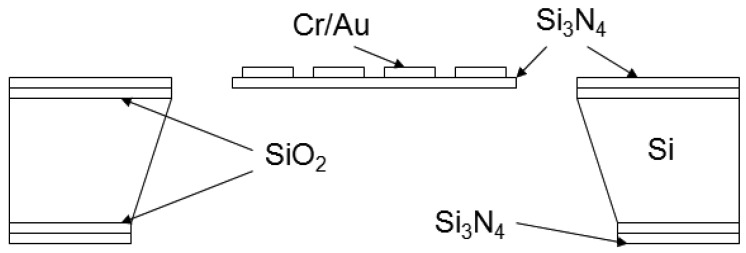
Cross-section of a finished bulk micromachined bridge or cantilever-type Pirani gauge.

**Figure 2.**
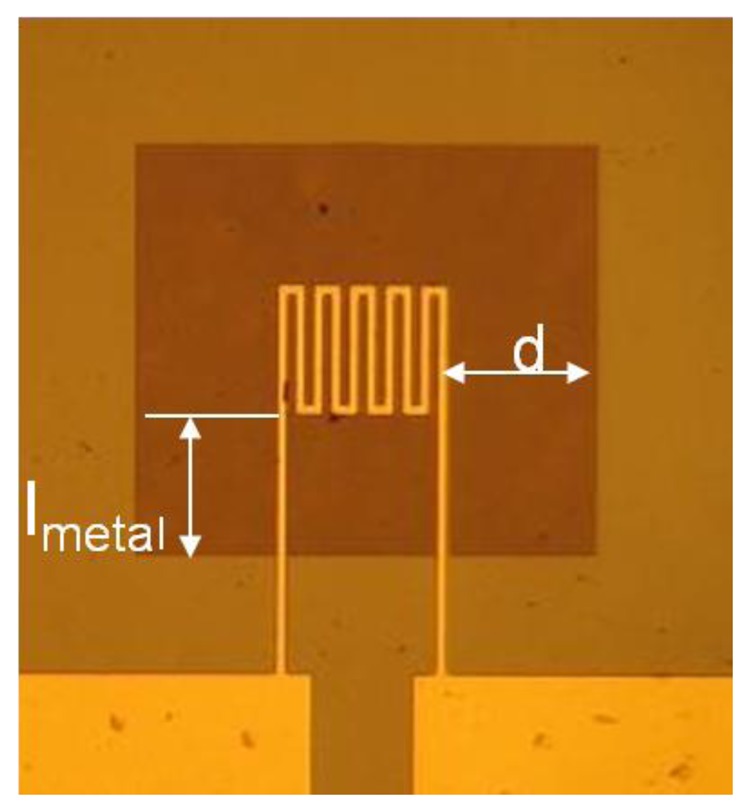
Optical micrograph of the top view of a 1,040 μm square silicon nitride membrane Pirani gauge with d the distance between the hot and cold region and *l_metal_* the length of the metal line connecting the hot to the cold regions.

**Figure 3.**
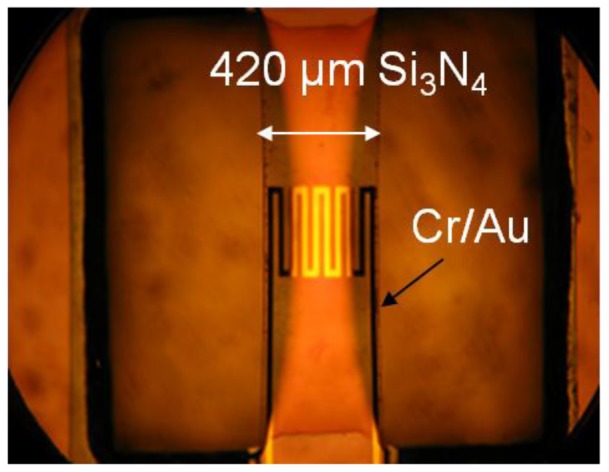
Optical micrograph of the top view of a bridge-type Pirani gauge. The silicon nitride bridge dimensions are 420 μm × 1,250 μm.

**Figure 4.**
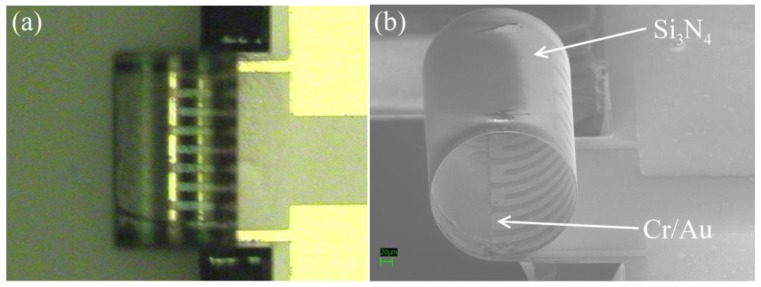
(**a**) Optical micrograph of an out-of-plane (curled) 420 μm × 800 μm cantilever-type Pirani gauge. (**b**) SEM image of the same type of gauge.

Figure 1 shows a schematic drawing of the cross-section of a finished cantilever or bridge device. The Cr/Au lines are drawn as one cross section because the Cr is only 20 nm thick and serves as an adhesion layer for the gold layer. Figure 2 shows an optical micrograph of a 1,040 μm × 1,040 μm membrane device in which the distance ‘d’ from the hot region to the silicon cold junction is 320 μm. The darker square region in the center is the silicon nitride diaphragm. The length of one section of the meander resistor element is 260 μm, not including the 20 μm width of the connecting elements. Figure 3 shows the optical micrograph of a bridge device with d = 450 μm. Figure 4 shows the optical micrograph (a) and SEM image (b) of an out-of-plane (curled) cantilever device.

No buckling of the membrane is observed in Figure 2 indicating that it is under tensile stress. Some deformation of the silicon bridge in Figure 3 near the center is observed. This is attributed to a built-in stress gradient in the nitride. The curled device in Figure 4 does not show that deformation perpendicular to its long axis which is attributed to stress release. This was also observed when some bridge devices severed at one end and formed curled cantilever devices similar to Figure 4.

## 3. Theoretical Considerations

From initial experiments, it was observed that inter-diffusion of the Cr/Au metallization took place when the meander resistor was heated above 180 °C. This manifested itself by an increase in resistance and reduction in the temperature coefficient of resistance (TCR) from 2,100 ppm/K to 600 ppm/K. Thus, the operating power of the devices was chosen so that this critical temperature was not reached at any pressure. For this reason, we have chosen to operate all of our devices in constant voltage mode. In one example discussed below, it will be shown that higher sensitivity is reached when operating in constant current mode.

### 3.1. Constant Voltage Operation

**Figure 5.**
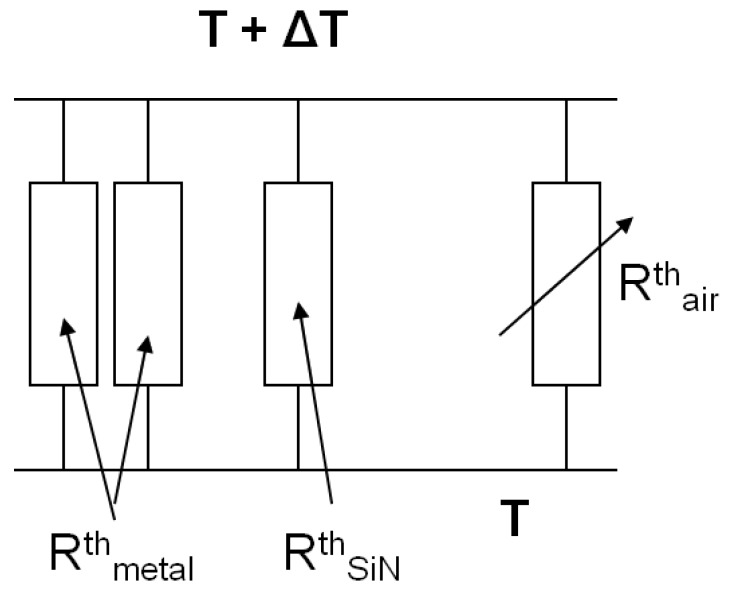
Equivalent thermal resistance network of the Pirani gauges.

Figure 5 shows the equivalent thermal resistance network adapted for our devices [14]. In thermal equilibrium, the thermal resistance *R^th^* is given by:

(1) $$\overset{1}{\;}/\underset{R^{th}}{\;}\;=\;\overset{2}{\;}/\underset{R_{\text{metal}}^{th}}{\;}\;+\;\overset{1}{\;}/\underset{R_{\text{SiN}}^{th}}{\;}\;+\;\overset{1}{\;}/\underset{R_{\text{air}}^{th}}{\;}$$

(2) $$\overset{1}{\;}/\underset{R^{th}}{\;}\;=\;\overset{P}{\;}/\underset{\Delta T}{\;}$$

where $R_{\text{metal}}^{th}$ is the thermal resistance of the two metal lines conducting heat from the periphery of the meander to the edge of the silicon nitride support structure. $R_{\text{SiN}}^{th}$ is the thermal resistance of the membrane, bridge or cantilever support. $R_{\text{air}}^{th}$ is the thermal resistance of air where heat is carried away from the resistor element through the air to the cold junction. $R_{\text{metal}}^{th}$ and $R_{\text{SiN}}^{th}$ are independent of pressure and only $R_{\text{air}}^{th}$ varies with pressure. P is the power dissipated by the resistor element and *ΔT* is the corresponding equilibrium temperature change of the resistor element at a given pressure *p*.

For constant voltage operation, the power is given by:

(3) $$P=\frac{V^{2}}{R}$$

and, following the derivation in [9], the resistance of the meander-shaped resistor is given by:

(4) $$R={\left(\alpha R_{0}R^{th}V^{2}+{\left(\overset{R_{0}}{\;}/\underset{2}{\;}\right)}^{2}\right)}^{\overset{1}{\;}/\underset{2}{\;}}\;+\;\overset{R_{0}}{\;}/\underset{2}{\;}$$

At pressures below 1 mTorr, $R_{\text{air}}^{th}$, for the devices presented here, becomes much larger than $R_{\text{metal}}^{th}$ and $R_{\text{SiN}}^{th}$. Heat is mostly conducted through the two resistor lines going from the hot region to the cold junction and along the silicon nitride diaphragm, bridge or cantilever. Thus:

(5) $$\overset{1}{\;}/\underset{R_{lp}^{th}}{\;}\;=\;\overset{P_{lp}}{\;}/\underset{\Delta T_{lp}}{\;}\;=\;\overset{2}{\;}/\underset{R_{\text{metal}}^{th}}{\;}\;+\;\overset{1}{\;}/\underset{R_{\text{SiN}}^{th}}{\;}$$

where *lp* stands for low pressure, and:

(6) $$\overset{1}{\;}/\underset{R^{th}}{\;}\;=\;\overset{P_{lp}}{\;}/\underset{\Delta T_{lp}}{\;}\;+\;\overset{1}{\;}/\underset{R_{\text{air}}^{th}}{\;}$$

Substituting Equation (6) into Equation (4) yields:

(7) $$R=\frac{R_{0}}{2}+{\left[{\left(\frac{R_{0}}{2}\right)}^{2}+\left(\frac{\alpha V^{2}R_{0}}{\overset{P_{lp}}{\;}/\underset{\Delta T_{lp}}{\;}\;+\;\overset{1}{\;}/\underset{R_{\text{air}}^{th}}{\;}}\right)\right]}^{0.5}$$

Here, *R*_0_ is the resistance of the heated resistor element measured at low power to avoid heating and α is the temperature coefficient of resistance (2.1 × 10^−3^ K^−1^) of the Cr/Au resistor element. For the devices presented, *R*_0_ = 40 Ω. The temperature rise *ΔT_lp_* is obtained from:

(8) $$\Delta T_{lp}=\frac{R_{lp}-R_{0}}{\alpha R_{0}}$$

where *R_lp_* is the resistance of the heated resistor element at the lowest pressure. The expression for $R_{\text{air}}^{th}$ is given in [9] using the formalism developed in [6]. *R* and *R*_0_ are electrical resistances with dimensions of ohms, whereas thermal resistances are denoted with the superscript, *th*, and have dimensions of K/W.

By measuring in constant voltage mode, the power is reduced at low pressures. This results in a reduced resistance change due to reduced heating and thus reduced sensitivity. The opposite is true when the gauge is operated in constant current mode. The power increases with decreasing pressure resulting in an increased change in resistance.

### 3.2. Constant Current Operation

The constant current mode is usually the preferred mode when measuring micromachined Pirani gauges. As mentioned above, we have chosen the constant voltage mode for most of our measurements to avoid possible excessive heating of the resistor element at low pressures which might cause inter-diffusion of Cr/Au, thus causing a change in the thermal coefficient of resistance α. For completeness, one measurement performed in constant current mode is included to demonstrate the higher sensitivity of this mode of operation.

The power is given by:

(9) $$P=I^{2}R$$

Using Equations (2), (8) and (9), the resistance of the heating element is given by:

(10) $$R=\frac{R_{0}}{1-R^{th}I^{2}R_{0}\alpha }$$

## 4. Testing

The devices were horizontally positioned according to Figure 1 and tested in a high vacuum test chamber equipped with three XYZ positioning probes, a microscope, a sample stage with heating capability to measure *α*, and a needle valve for manual air intake. The pressure in the chamber was monitored with three pressure gauges: a thermocouple gauge measuring pressure in the range of 1 Torr–760 Torr, another thermocouple gauge monitoring pressures from 1 mTorr to 1 Torr, and an ion gauge capable of sensing pressures from 10^−9^ Torr to 1 mTorr. Contact to the resistor pads was made with copper probes positioned at the end of two of the XYZ manipulators.

To obtain the resistance as a function of pressure in constant voltage mode, a power of 2 mW was applied to the device at atmospheric pressure and the chamber was pumped down to 0.1 mTorr. Using the needle valve, the pressure in the chamber was increased to desired values and the corresponding current was recorded. Resistance R is obtained by dividing voltage over current. In constant current mode, the voltage across the resistor element was monitored as a function of pressure and resistance R, again, was obtained by dividing voltage over current.

Care was taken that the distance between the resistors and the metal platform in the vacuum system was larger than the largest ‘d’. This was accomplished by placing the wafer on two copper blocks containing the test devices between the blocks. Without this precaution, some ambiguity in the results might occur due to a ‘d’ that might be smaller than the ‘d’ of the device and thus alter the results.

## 5. Experimental Results

### 5.1. Resistance *versus* Pressure

**Figure 6.**
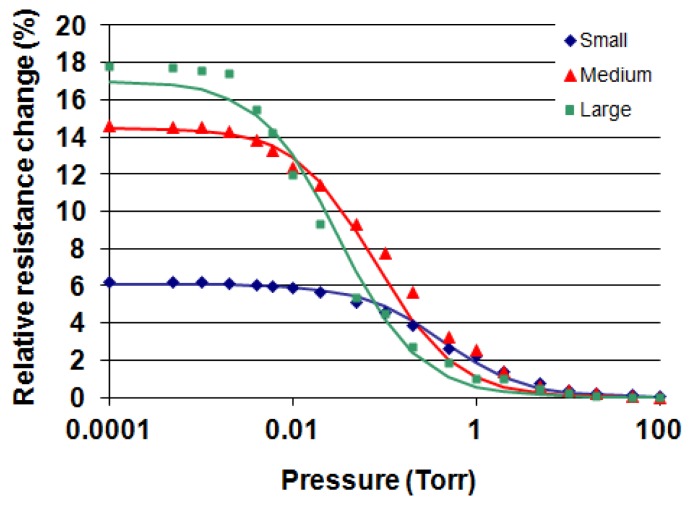
Relative resistance change (%) of a small (470 μm), medium (1,040 μm) and large (2,740 μm) membrane Pirani gauge as a function of pressure. The power applied to the heating elements at 760 Torr was 2 mW. The devices were measured in constant voltage mode. The solid curves are calculated values.

Figure 6 shows the relative resistance change *ΔR/R* (%) *versus* pressure for a small, medium and large membrane device operated at 2 mW at atmospheric pressure. Resistance changes of about 6%, 14% and 18% are observed over the measured pressure range. *R* is the resistance of the powered gauge at 760 Torr. The increase in resistance as a function of membrane size is entirely caused by the increase in $R_{\text{metal}}^{th}$ and $R_{\text{SiN}}^{th}$. The midpoints of resistance change (maximum sensitivity) are at approximately 0.3 Torr, 0.15 Torr and 0.02 Torr, respectively and are caused by increasing d's of about 65 μm, 350 μm and 1,200 μm, respectively. The solid lines are calculated values using the equations given above. From Equation (5) and calculating:

(11) $$R_{\text{metal}}^{th}=\frac{l_{\text{metal}}}{k_{\text{metal}}w_{\text{metal}}t_{\text{metal}}}$$

**Figure 7.**
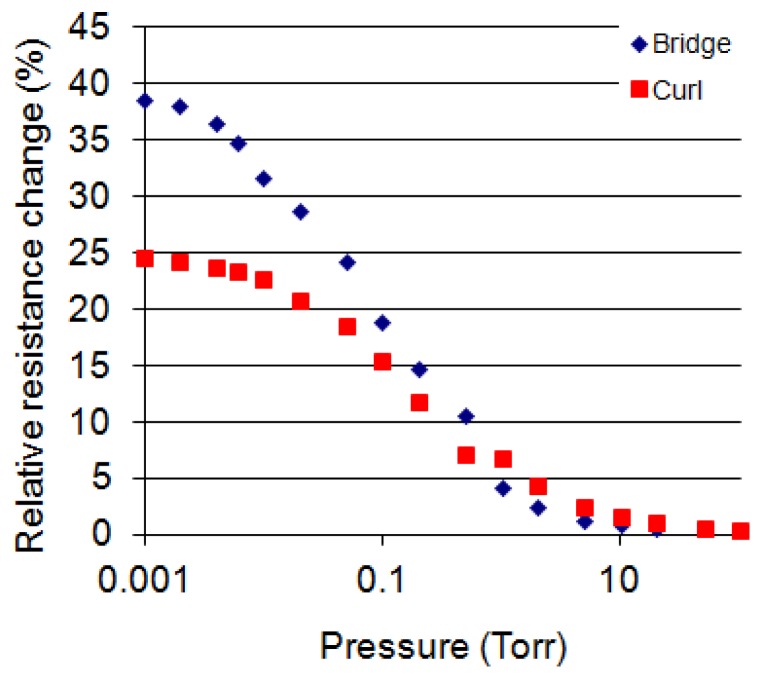
Relative resistance change (%) *versus* pressure of a curl device (bottom) and of a bridge device (top). The power applied to the heating elements at 760 Torr was 2 mW. The devices were measured in constant voltage mode.

$R_{\text{SiN}}^{th}$ is obtained. Figure 7 shows *ΔR/R* (%) *versus* pressure curves for a bridge device and an out-of-plane cantilever device measured at 2 mW. The relative resistance changes of these two devices are significantly larger than the membrane devices. This is caused by increased thermal resistances as compared to the membranes. When designing these two gauges, it was expected that the cantilever device would exhibit the largest resistance change due to maximum $R_{\text{SiN}}^{th}$ since only one thermal path is available to carry heat away. However, its resistance change is lower than the bridge device. This is caused by a thermal shunt where some of the heat is conducted from the curled distal end of the cantilever touching the lower portion of the curl. Figure 7 also shows that the curled device is more sensitive at pressures ranging from 0.2 Torr tp 5 Torr as compared to the bridge device; the reason being that its ‘d’ is distributed from about d = 30 μm to 450 μm.

Table 1 summarizes some of the results. $R_{\text{metal}}^{th}$ is obtained from Equation (11) using *k_metal_* = 320 W/m·K [14], *l_metal_* for the different devices, *w_metal_* = 20 μm and *t_metal_* = 0.2 μm. $R_{\text{SiN}}^{th}$ is obtained from Equations (5) and (8). The pressure *p* at highest sensitivity is at the midpoint of the resistance *versus* pressure curve. The range is defined as the ratio of pressures where *ΔR/R* is 10% and 90%, respectively.

From Table 1 it is observed that *ΔR/RP* increases with *R_lp_* for the different devices and that the range varies from 200 to 375 for the three membranes and the bridge device, whereas that of the curl device is 750. $R_{\text{air}}^{th}$ at 0.0002 Torr is about 1 × 10^7^ K/W which is larger than the largest $R_{lp}^{th}$ of 1.2 × 10^5^ K/W, thus justifying the assumption made in Equation (5).

### 5.2. Extension of Pressure Operating Range

The pressure range of the curl device is larger compared to the other devices with fixed d's. Some of the devices presented in the literature also have different d's. For instance, the meander device in [8] is fabricated over a 1 μm gap, but also has a 100 μm gap from the meander-shaped resistor to the silicon substrate. The 1 μm gap is responsible for the enhanced sensitivity at higher pressures. In [3] it is shown that by placing a silicon cover (micro cap) with different d's, which are smaller as compared to the device's ‘d’, over the resistor element, the pressure range is extended towards higher pressures.

**Figure 8.**
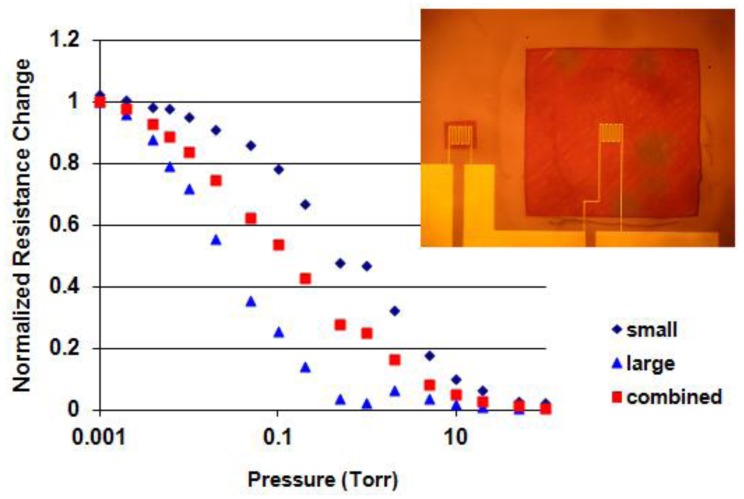
Normalized resistance change *versus* pressure for a small and large membrane device and for a combined device where both membranes were connected in series. The insert shows an optical micrograph of the top view of the devices. Not shown is the 110 Ω shunt resistor that was externally connected in parallel to the large membrane device to match the resistance change of the smaller membrane device.

Another approach is to combine at least two devices with different d's. This concept is illustrated with two membrane devices with d = 65 μm and d = 1,200 μm placed next to each other on a chip and connected in series. Figure 8 shows the results. The normalized relative resistance changes are plotted as a function of pressure for the two individual devices and for the combined device. The insert shows the two devices connected in series. From Figure 6 it can be seen that the smallest membrane device is not as sensitive as the largest device since the largest device has higher thermal resistances. By putting those two devices in series, the combined device would be very similar to the largest device. In order to cover the entire pressure range of the individual devices, their relative resistance change should be equal. This was accomplished by connecting an external shunt resistor of 110 Ω across the larger device. Whereas the ranges of the individual devices are 375 for the small membrane and 330 for the large membrane, that of the combined device is 800.

### 5.3. Improving Sensitivity

A measure of sensitivity is the maximum resistance change between high and low pressures. From Table 1, changes ranging from 3%/mW to 19%/mW are obtained. The difference is entirely due to the increased thermal resistances of the bridge device over the three membrane devices.

**Figure 9.**
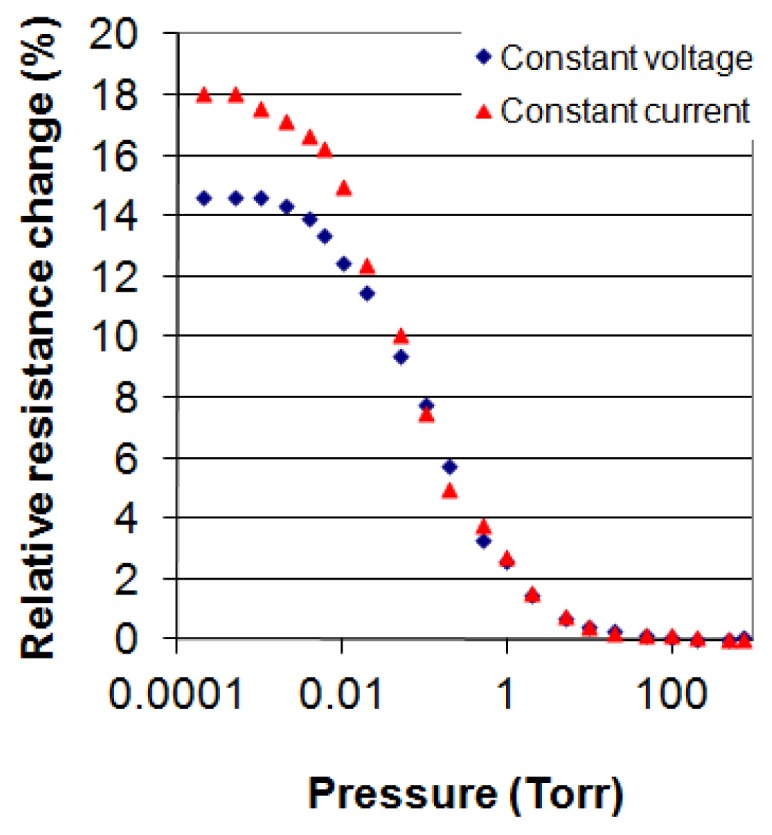
Relative resistance change *versus* pressure of a medium membrane device measured at about 2 mW in constant voltage (lower curve) and in constant current (upper curve) modes.

Another way to increase sensitivity is by operating the device in constant current mode. This is illustrated in Figure 9. It shows results of a medium size membrane device (Figures 2 and 6), operated at constant voltage and constant current. Whereas in the constant voltage mode the maximum relative resistance change is 14.6%, it is 18% in the constant current mode, an increase of 23%. The device was measured by applying a power of about 2 mW at 760 Torr. In constant voltage mode, the power decreased from 2.1 mW at 760 Torr to 1.87 mW at 0.0002 Torr and increased in the constant current mode from 2 mW at 760 Torr to 2.35 mW at 0.0002 Torr. In constant voltage mode, the temperature of the sensor increased from 20 °C to 90 °C above ambient over the measured pressure range and from 20 °C to 110 °C in constant current mode. The temperature rise in constant current mode is still below the inter-diffusion temperature of Cr/Au and thus it is safe to operate the device in constant current mode at a power of 2 mW. The temperature increases were calculated using Equation (8) and substituting *R_lp_* with *R*.

## 6. Conclusions

Meander-shaped Cr (20 nm)/Au (200 nm) resistors with a surface area of 6.3 × 10^−4^ cm^2^ were fabricated on 1 μm thick, LPCVD deposited, silicon nitride membranes, bridges and cantilevers using silicon bulk micromachining. At atmospheric pressure (760 Torr) a constant voltage was applied to the resistors and the current was adjusted so that about 2 mW of power was dissipated causing the resistors to heat to several degrees above room temperature. With decreasing pressure, the temperatures of the resistors increased due to increasing thermal resistance of air.

It is shown that the thermal resistances of the metal lines connecting the meanders to the edge of the silicon wells and of the silicon nitride structures determine the magnitude of the resistance changes with the small membrane device exhibiting the lowest resistance change and the bridge device the largest. As long as the Cr/Au resistors are not heated above 180 °C at low pressures, it is demonstrated that operating the devices in constant current mode might be preferred since the resistance change is about 20% higher compared to the constant voltage mode.

It is also shown that the resistance-*versus*-pressure curves follow an inverted S-shape and that the midpoint of that curve depends on the distance ‘d’ between the heated resistor and the silicon cold junction. Depending on ‘d’, the curves are shifted parallel along the log pressure scale. Each individual device has a range of about 200–350. Range here is defined as the ratio of pressures between the 10% point of resistance change and the 90% point.

By putting two membrane devices with different d's in series, it is shown that the range of the combined device can be increased significantly to about 800 for the given example. This concept can be extended to more than two devices. Due to stress gradients in the silicon nitride, the cantilever devices, which were supposed to exhibit the largest resistor changes, formed out-of-plane devices in the shape of cylinders. Since touching of the distal end of the silicon nitride occurs near the silicon well, the devices act like distributed ‘d’ devices and exhibit a range of 750, similar to the two membrane devices in series. Even though a thermal shunt diminishes the resistance change to 10.5%/mW, it is larger than the two membranes put in series. The two membrane configuration exhibits a resistance change of about 3%/mW. This is caused by the relative small thermal resistances of the small membrane and the need of shunting the larger, more sensitive device. An improvement of this concept can be obtained by designing the different ‘d’ devices with equal thermal resistances.

## Figures and Tables

**Table 1.** Device and performance parameters for the three membranes, the bridge and cantilever devices. The thermal resistances of the metal and silicon nitride of the curled device cannot be obtained using the procedure followed for the other devices due to thermal shunting.

**Device**	**Nitride dimensions** **(μm)**	${\text{R}}_{\text{metal}}^{\text{th}}$ **(K/W)**	${\text{R}}_{\text{SiN}}^{\text{th}}$ **(K/W)**	${\text{R}}_{\text{lp}}^{\text{th}}$ **(K/W)**	***ΔR/RP*** **(%/mW)**	***p* at highest sensitivity** **(Torr)**	**Range** **p(*ΔR/R* at 10%)/** **p(*ΔR/R* at 90%)**
Small membrane	470 × 470	6.8 × 10^4^	4.6 × 10^4^	1.9 × 10^4^	3.1	0.3	375
Medium membrane	1,040 × 1,040	2.9 × 10^5^	7.3 × 10^4^	4.9 × 10^4^	7.3	0.15	330
Large membrane	2,740 × 2,740	9.6 × 10^5^	6.9 × 10^4^	6.0 × 10^4^	8.9	0.02	330
Bridge	420 × 1,250	3.7 × 10^5^	3.7 × 10^5^	1.2 × 10^5^	19	0.1	200
Curl	420 × 800				10.5	0.2	750
